# Use of a self-perception questionnaire for screening auditory abilities in children with behavioral dysphonia

**DOI:** 10.1590/2317-1782/e20230314en

**Published:** 2025-02-10

**Authors:** Ana Carolina Pinto Lemos, Tamy Nathalia Tanaka, Ana Carolina Constantini, Rebecca Christina Kathleen Maunsell, Maria Isabel Ramos do Amaral

**Affiliations:** 1 Curso de Fonoaudiologia, Departamento de Desenvolvimento Humano e Reabilitação, Faculdade de Ciências Médicas – FCM, Universidade Estadual de Campinas – UNICAMP - Campinas (SP), Brasil.; 2 Curso de Medicina, Departamento de Oftalmologia e Otorrinolaringologia, Faculdade de Ciências Médicas – FCM, Universidade Estadual de Campinas – UNICAMP - Campinas (SP), Brasil.

**Keywords:** Auditory Perception, Child, Screening, Questionnaire, Hearing Tests

## Abstract

**Purpose:**

To discuss the use of a self-perception questionnaire on auditory abilities applied to children with behavioral dysphonia and compare it with the perception of auditory and voice symptoms, as well as with performance in temporal tests of auditory processing.

**Methods:**

17 children, aged 6–8 years, with a diagnosis of behavioral dysphonia. Individuals with peripheral hearing loss, severe visual and/or language impairments or neurodevelopmental disorders were excluded. The following instruments were applied: pediatric voice symptoms questionnaire (PVSQ, brazilian validated version); questionnaire of self-perception auditory skills (QAPAC) inserted into the online program AudBility with its self-assessment and parental versions; basic audiological evaluation and the temporal tests Random Gap Detection (RGDT), and Frequency Pattern (FPT). Parents’ and children’s responses were compared and Spearman’s correlation measured correlation between the QAPAC and the PVSQ, as well as between questionnaires and temporal tests.

**Results:**

QAPAC self-assessment version showed a mean score of 45.5±7.4, wherein seven (41.2%) children scored below the risk criteria for Central Auditory Processing Disorder (CAPD). The mean score on the parental version was 39.5±10.5, with 11 (64.7%) responses falling below the risk criteria. Parents’ mean score was statistically lower (worse) compared to that of the children (p<0.005). A strong correlation was found between the self-assessment versions of QAPAC and PVSQ (r=0.671), alongside the parental versions (r=0.722). A poorer performance of the left ear in comparison to the right ear was observed in the FPT test (p<0.005), and a moderate correlation between QAPAC and FPT in the left ear during the imitation phase was noted (r=0.597).

**Conclusion:**

The use of self-perception questionnaire on auditory abilities is a valid contribution to initial voice assessment in children with behavioral dysphonia.

## INTRODUCTION

Central auditory processing disorder (CAPD) is characterized by the presence of alterations in one or more auditory skills and has direct negative impacts on the development of a child’s communication and learning skills^(1)^. The diagnosis of CAPD is obtained from a battery of special behavioral tests that assess each of these skills, and can be complemented by questionnaires and/or objective electrophysiological tests^(2)^.

Considering the high incidence of CAPD in school-age children^(3)^, screening protocols are extremely important, as they identify children at risk for CAPD. Then, an assertive referral is made for diagnostic evaluation. Once the diagnosis is made, early rehabilitation can be performed to minimize future losses minimized^(4)^. AudBility^(5)^, an online auditory processing screening program, was recently developed and, since then, studies have demonstrated its feasibility of application and validation as a screening instrument^(6-8)^. The program has different modules, depending on the age group to which it will be applied. Every module has auditory tasks, and the questionnaire of self-perceived auditory skills (QAPAC), which can be answered by the subject undergoing screening, their parents or teachers^(6-8)^.

Clinical guidelines recommend the use of checklists and/or self-perception questionnaires, such as QAPAC, in CAP screening, since they are simple, accessible, easy-to-apply resources^(2,9)^. In addition, the questionnaires focus on the subject, identifying the daily difficulties faced by them and their caregivers. A study found a positive correlation between the QAPAC and the “Simplified Auditory Processing Assessment” (ASPA) in a sample of children with and without school difficulties, regardless of the version applied (self-assessment or parental assessment)^(10)^. ASPA refers to a simple battery of tests for auditory skills, commonly performed in Brazil because it is a low-cost and fast protocol that analyzes sound localization and memory skills for verbal and nonverbal sounds in sequence through diotic (open field) tasks with sound instruments. These results confirmed the complementarity of the questionnaire and a battery of tests for auditory skills^(10)^.

To date, AudBility has been validated as a screening protocol for children with typical development^(7)^. In the validation study, the program was applied to 154 children with good school performance. Of these, 112 also underwent the diagnostic behavioral assessment of central auditory processing (CAP). The results showed accuracy values ranging from 54.1% to 84.4%, and AudBility was considered an effective screening instrument for auditory skills. Based on this validation, its contribution when applied to other populations should be investigated. Among the changes associated with CAPD, behavioral dysphonia is a voice deviation that occurs due to inappropriate use of the voice or inadequate vocal technique, making it difficult to produce natural sound and negatively affecting the subject’s communication^(11)^.

Evidence suggests that voice complaints may be related to CAP difficulties and indicates changes in the temporal auditory skills of dysphonic children^(12,13)^. Temporal auditory skills, especially those of temporal ordering and resolution, are involved in voice processing, and it is known that an individual with difficulty processing the frequency, intensity, and duration of speech has impaired self-monitoring and, consequently, impaired vocal production^(14,15)^.

It is believed that screening the auditory processing of children with behavioral dysphonia would allow simple and fast identification of children who could benefit from a complete diagnostic evaluation of CAP. Therefore, this study aimed to describe and analyze the use of the questionnaire of self-perceived auditory skills applied to children with behavioral dysphonia and compare it to the perception of auditory and voice symptoms and to the performance in temporal tests of auditory processing.

## METHODS

### Study design, study site, and ethical aspects

This is a quantitative descriptive prospective cross-sectional study, approved by the Research Ethics Committee, under report 4.793.214. This study is part of a larger project named “Applicability of the auditory skills screening program – AudBility – in children with behavioral dysphonia.” Data collection took place at the otorhinolaryngology, voice and audiology services of the institution where the study was conducted. Participation was voluntary, and the informed consent form was signed by the parents and the informed assent form was signed by the children.

### Participants and selection criteria

The subjects were selected after the application of a survey of the demand for children with behavioral dysphonia, aged 6 to 10 years, of both sexes, who were admitted to the service through the vocal emergency care of the institution and a basic health facility in the municipality.

This study included children who were native speakers of Brazilian Portuguese with a confirmed diagnosis of behavioral dysphonia based on medical and speech-language evaluations. Children with peripheral hearing loss, a history of recurrent otitis media, severe visual changes, cognitive changes/syndromes and/or neurodevelopmental or language disorders, and anatomical changes in the larynx were excluded. Children who had already undergone speech therapy for vocal and/or language disorders and who had school difficulties attested by the teacher and confirmed by the history collected from parents and/or guardians were also excluded.

### Procedures

Data collection was performed in two stages. The first stage consisted of confirming the diagnosis of behavioral dysphonia and normal peripheral hearing function through an otorhinolaryngological and speech-language evaluation, in addition to assessing good school performance. These procedures are described below:

- Speech-language assessment of the voice: the voice was recorded in a soundproof booth and an auditory-perceptual assessment of the vocal quality was performed by two neutral judges, speech therapists with experience in voice analysis. For this assessment, the Consensus Auditory-Perceptual Evaluation of Voice (CAPE-V) visual analog scale was used^(16)^. As part of the speech-language assessment of the voice and for data collection, the Pediatric Voice Symptoms Questionnaire (PVSQ – Brazilian Validated Version)^(17)^, was applied, in the version answered by the child (self-assessment) and the version answered by the parent. In the PVSQ, the questions involve the use of the voice and the impacts of vocal changes. The four domains of voice are addressed: singing, speaking, shouting, and voice projection, and participants answer about the frequency of each situation presented (0=never, 1=sometimes, 2=almost always, and 3=always). The maximum score is 38, which indicates high impact. The cutoff value that indicate vocal changes are: 2.1 for the parental version and 7.6 for the self-assessment version. Data obtained in the PVSQ, in addition to being used in the vocal assessment, were analyzed and compared with the results found in the QAPAC and in the behavioral tests of temporal auditory processing.
- Otorhinolaryngological evaluation: performed by an otorhinolaryngologist specializing in pediatric care. During the medical evaluation, data on respiratory and audiological complaints were collected and an otorhinolaryngological physical examination was performed, including video nasolaryngoscopy.
- Basic audiological assessment: consisted of otoscopy, anamnesis, pure tone audiometry, speech audiometry, and immittance audiometry to confirm hearing within normal standards (OMS, 2014) and a type A tympanometric curve^(18)^. Children with excess earwax were referred for removal and later recalled. The tests were performed with a GSI AudioStar Pro audiometer in a soundproof booth and with an Interacoustics AT235 ear analyzer and audiometer, both properly calibrated.
- Confirmation of good school performance: a questionnaire was sent to the child’s teacher addressing the student’s school performance, perception of auditory and attentional behavior, and relationship with peers. This questionnaire was given to the parents on the first day and brought back answered by the teacher on the second day of data collection. In addition to the questionnaire answered by the teacher, the absence of school complaints reported by the parents or guardians at the time of history taken was also considered.

The second stage, performed on another day, consisted of the application of the AudBility program questionnaire and diagnostic tests of temporal auditory processing in a soundproof booth.

The AudBility program consists of the questionnaire of self-perceived auditory skills (QAPAC), and a battery of auditory tasks. In this study, only the responses to the QAPAC were analyzed, as detailed below:

- Questionnaire of self-perceived auditory skills (QAPAC)^(10)^: The QAPAC was developed based on the Scale of Auditory Behaviors (SAB)^(19)^ and published in 2018^(10)^, in self-assessment and parental versions, available in Chart 1. Twelve direct questions are presented addressing auditory behaviors in quiet and noisy environments, in a language that is understandable to the participants, who answer at what frequency a certain auditory behavior occurs in each situation. Before the question, there is an example situation that contextualizes the question for a better understanding. Each answer receives a score according to the Likert scale: always (1), frequently (2), sometimes (3), rarely (4), and never (5). The result is calculated by adding up the scores of the answers, and the final score can range from 12 to 60. Scores under 45 were considered at risk for the occurrence of CAPD^(6,7,19)^.

**Chart 1:** QAPAC questions – self-assessment and parental versions

**Questionnaire – child**	**Questionnaire – parent**
**You are in the classroom or in an environment where people are talking,**	**When your child is in an environment where people are talking,**
**1. Do you have difficulty listening or understanding what the teacher or someone else is saying?**	**1. Does he/she have difficulty listening or understanding what the people are saying?**
**The teacher or someone else is talking too fast to you,**	**If you talk too fast to your child,**
**2. Do you have difficulty understanding what the teacher just said?**	**2. Does he/she have difficulty understanding what you just said?**
**The teacher or someone else is giving you spoken instructions (explanations),**	**When you give spoken instructions (explanations) to your child,**
**3. Do you have difficulty following spoken instructions?**	**3. Does he/she have difficulty following spoken instructions?**
**The teacher or someone else is talking to you in a quiet environment,**	**You are talking to your child in a quiet environment,**
**4. Do you have difficulty listening and understanding the words clearly without changing any letter?**	**4. Does he/she have difficulty listening and understanding the words clearly without changing any letter?**
**When the teacher or someone else is talking to you,**	**When you are talking to your child,**
**5. Do you feel that sometimes you hear well and sometimes you don’t?**	**5. Do you feel that sometimes he/she hears well and sometimes he/she doesn’t?**
**You are in the classroom or the schoolyard and someone calls your name,**	**When your child is called by his/her name in a large place,**
**6. Do you have difficulty understanding where the sound is coming from?**	**6. Does he/she have difficulty understanding where the sound is coming from?**
**The teacher or someone else is talking to you,**	**When you are talking to your child,**
**7. Do you ask this person to repeat what he or she said?**	**7. Does he/she ask you to repeat what was said?**
**You are in the classroom,**	**When your child is at home or in other environments,**
**8. Do you get distracted easily?**	**8. Does he/she get distracted easily?**
**Last year at school,**	**Last year,**
**9. Did you have learning difficulties?**	**9. Did he/she have learning difficulties?**
**You are doing an activity,**	**When your child is doing a school activity,**
**10. Do you have trouble focusing?**	**10. Does he/she have trouble focusing?**
**When you are in the classroom or at home,**	**When your child is at home,**
**11. Do people tell you that you are daydreaming or inattentive?**	**11. Do you think he/she is daydreaming or inattentive?**
**When you are at school or at home,**	**When your child is at home,**
**12. Are you disorganized?**	**12. Is he/she disorganized?**

**Figure 1:**
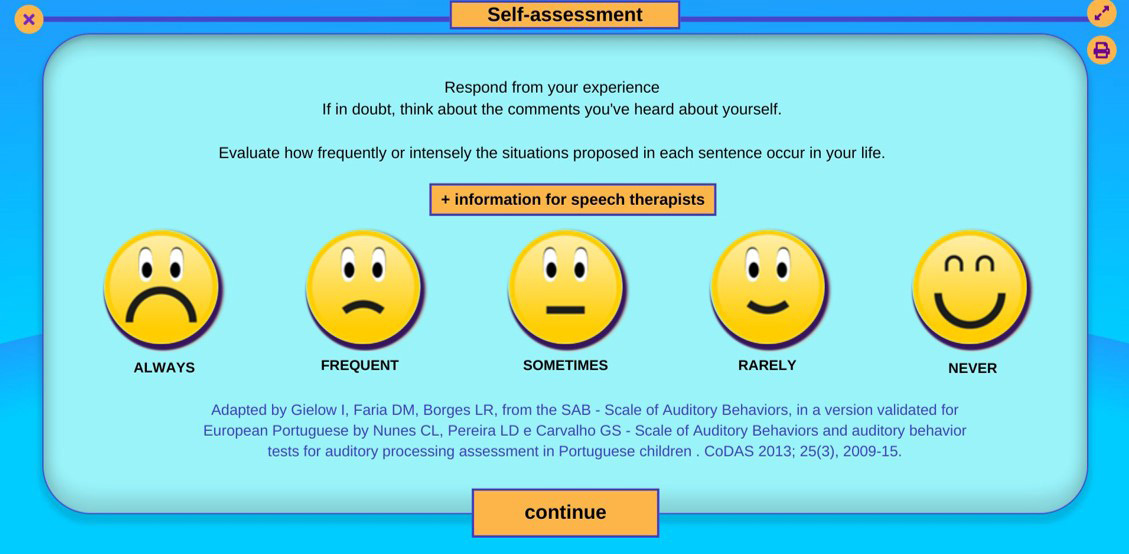
QAPAC training screen

**Figure 2:**
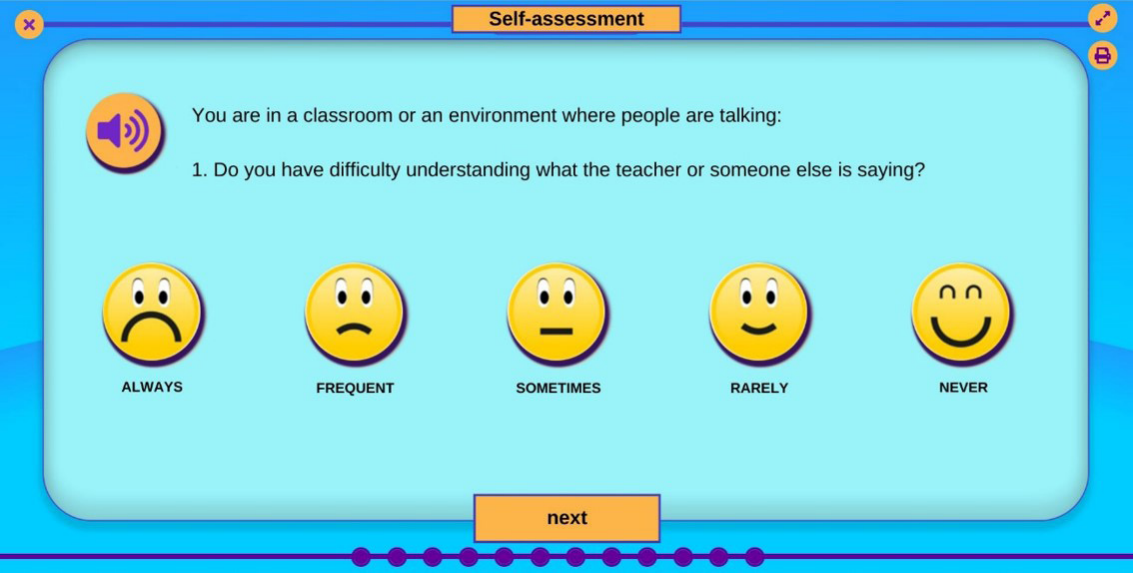
AudBility program screen, first question of the QAPAC

The program displays an introduction screen with the questionnaire and the answer options, as well as pictograms related to each of the answer options to help the child understand, as illustrated in Figure 1. Figure 2 shows the screen with the first question of the QAPAC, with the answer options.

The researcher read the questions together with the child to help him/her perform the task. The child indicated his/her answer on the computer screen and the researcher selected the desired option.

- Behavioral assessment of Temporal Auditory Processing: Behavioral tests of temporal auditory processing were performed in a soundproof booth, with a GSI AudioStar Pro audiometer and supra-aural headphones that were properly calibrated. The tests are described below:
  - Random Gap Detection Test (RGDT)^(20)^: It assesses the temporal resolution ability in a binaural task at an intensity of 50 dB SL (sensation level). Pairs of pure tones are presented at frequencies of 500, 1000, 2000, and 4000 Hz, with intervals/gaps between the two tones, of variable duration, and randomly arranged. The gaps have intervals of 0, 2, 5, 10, 15, 20, 25, 30, and 40 milliseconds (ms) and the test presents a training range. Children were instructed to respond by gestures whether they heard/perceived one or two tones, and it was possible to observe whether or not they noticed the presence of the interstimulus interval. The gap detection threshold, that is, the shortest perceived time interval, was determined. The threshold was calculated individually for each frequency, as well as the arithmetic mean of results at the four frequencies evaluated. The normality criterion considers a threshold less than or equal to 15 ms for children aged 6 years and less than or equal to 10 ms for children aged 7 years or older^(21)^.
  - Frequency Pattern Test (FPT): It assesses the temporal ordering ability in a monaural task, at an intensity of 40 dBSL (sensation level). It has 30 sequences of three tones that differ in frequency, in two different stages: imitation and naming of sounds (low or high). The Auditec of St Louis version^(22)^ was applied, which has a list of 30 sequences per ear in each stage that are combinations of pure tones of different frequencies – low: 880 Hz / high: 1430 Hz; with a duration of 500 ms. The percentage of correct answers was calculated per ear and the normality criterion considers a consider percentages greater than or equal to 60% for children aged six and seven years old, and greater than or equal to 81.5% for children aged eight years or older.

### Analysis of results

The statistical analysis used SPSS V20, Minitab 16, and Excel Office 2010 software tools. The variables was described using descriptive and inferential statistics.

The Equality of Two Proportions test analyzed the sample in relation to sex and distribution of the relative frequency (percentage) of the result “pass or fail” in the questionnaires. The Wilcoxon test compared the performance between the answers of the child (self-assessment) and of the parents (parental assessment) in each of the questionnaires applied (QAPAC and PVSQ) and the performance between the right and left ears in the frequency pattern test. Spearman’s correlation measured the degree of correlation between the PVSQ and the QAPAC, and between the questionnaires and the temporal tests (FPT and RGDT). The following scale was assumed for the correlation coefficients (r): 0.10 to 0.40 – weak correlation; 0.40 to 0.60 – moderate correlation; 0.60 to 1.00 – strong correlation. The confidence interval adopted was 95%, with a significance level of 5%. Significant findings (p<0.05) were highlighted in bold and with an asterisk (*) in the Tables.

## RESULTS

The sample consisted of 17 children, aged between 6 and 10 years, mean age: 7.8±1.5 years. A low variability was observed in age (CV=19%) and homogeneity between the sexes (p value=0.003), with 7 (41.2%) female participants and 10 (58.8%) male participants. Only two children (11.76%) were left-handed.

Regarding the sample performance in the questionnaires, the mean score in QAPAC was 45.5 in the self-assessment version and 39.5 in the parental version. The comparison between the answers of the versions showed a worse perception of the parents regarding the child’s auditory behavior (p=0.016). In addition, the mean score obtained in the application with the parents was below the cutoff score (<45), suggesting a risk for the occurrence of central auditory processing disorder (CAPD). In the PVSQ, the mean value is above the cutoff score in both versions: 11.1 in the self-assessment and 12 in the parental version, and, in this instrument, such data indicate the perception of voice symptoms. No significant difference was observed in the comparison between the answers of the PVSQ versions.

A significant difference was observed between the number of children who passed and failed the PVSQ (Table 1), in the self-assessment and the parental versions.

**Table 1:** Sample distribution in the two versions of the Questionnaire of Self-perceived Auditory Skills (QAPAC) and the Pediatric Voice Symptoms Questionnaire (PVSQ)

		**N**	**%**	**p-value**
**QAPAC – self-assessment**	Failed	7	41.2%	0.303
	Passed	10	58.8%	
**QAPAC – parent assessment**	Failed	11	64.7%	0.086
	Passed	6	35.3%	
**PVSQ – self-assessment**	Failed	12	70.6%	0.016*
	Passed	5	29.4%	
**PVSQ – parent assessment**	Failed	16**	100%	**<0.001** *
	Passed	0	0%	

* Test for Equality of Two Proportions;

** One parent/guardian did not answer the questionnaire

When correlated, the mean score of the sample obtained in the QAPAC in relation to the mean score obtained in the PVSQ (Table 2) showed a strong negative correlation between the versions of the questionnaires answered by the children and between the versions answered by the parents, i.e., the greater the frequency of voice symptoms, the greater the risk for CAPD.

**Table 2:** Correlation between the scores obtained in the Questionnaire of Self-perceived Auditory Skills (QAPAC) and the Pediatric Voice Symptoms Questionnaire (PVSQ) – self-assessment and parental versions

		**PVSQ (child)**	**PVSQ (adult)**
**QAPAC (child)**	**Corr** ** **(r)**	−0.671	−0.170
	**p-value**	0.003*	0.529
**QAPAC (adult)**	**Corr (r)**	−0.387	−0.722
	**p-value**	0.125	**0.002** *

* Spearman’s correlation;

** Corr: correlation

Of all 17 children in the sample, 14 attended the second day of data collection for the behavioral assessment of temporal auditory processing. Of all subjects evaluated in the RGDT, only one (7.14%) presented altered results. In the FPT, 9 (64.28%) children presented altered performance in at least one ear in one of the stages and 5 (35.71%) children presented normal results. Table 3 shows the average performance of the sample in the FPT and RGDT, and the comparison between the average performance of the right and left ears in the FPT. A statistical difference was observed in the naming stage, with better performance of the right ear (p<0.005).

**Table 3:** Sample performance in the frequency pattern test (FPT) and in the random gap detection test (RGDT) and comparison between the ears in the FPT

**Temporal auditory processing**		**N**	**Mean**	**Median**	**Standard deviation**	**CI**	**LE × RE**
**Imitation - FPT**	**RE**	14	74.5	88	28.1	14.7	0.374
	**LE**	14	77.4	80	21.3	11.1	
**Naming - FPT**	**RE**	14	64.1	62	16.8	8.8	**0.026** *
	**LE**	14	56.9	52	21.6	11.3	
**RGDT**		14	4,8	4	3	1.7	-

* Wilcoxon test

**Caption:** LE = left ear; RE = right ear; CI = confidence interval

Table 4 shows the correlations between the questionnaires studied (QAPAC and PVSQ) in relation to the two tests of temporal auditory processing. A moderate positive correlation was observed between the QAPAC in the self-assessment version and the FPT, considering the imitation stage of the left ear (p<0.005).

**Table 4:** Correlation between the Questionnaire of Self-perceived Auditory Skills (QAPAC), the Pediatric Voice Symptoms Questionnaire (PVSQ), and the temporal auditory processing tests

Temporal auditory processing		**PVSQ (child)**	**PVSQ (adult)**	**QAPAC (child)**	**QAPAC (adult)**
**RGDT**	Corr (r)	0.277	0.451	−0.093	−0.211
	p-value	0.384	0.164	0.774	0.510
**FPT – RE (naming)**	Corr (r)	−0.150	0.448	0.124	−0.354
	p-value	0.609	0.124	0.673	0.214
**FPT – LE (naming)**	Corr (r)	−0.209	0.532	0.254	−0.389
	p-value	0.472	0.061	0.380	0.170
**FPT – RE (imitation)**	Corr (r)	−0.214	0.351	0.506	−0.145
	p-value	0.463	0.240	0.065	0.621
**FPT – LE (imitation)**	Corr (r)	−0.509	0.055	0.597	0.179
	p-value	0.063	0.858	**0.024** *	0.541

* Spearman’s correlation.

**Caption:** LE: left ear; RE: right ear; FPT: frequency pattern test

Finally, Table 5 shows that, of all 14 children evaluated in the temporal auditory processing tests, 9 had alterations. Of these 9 children, 7 presented a risk for CAPD in at least one of the versions of the QAPAC.

**Table 5:** Individual conditions of the sample in relation to the temporal auditory processing tests and the questionnaire of self-perception o central auditory processing (QAPAC)

**Subjects**	**QAPAC – self-assessment**	**QAPAC – parent assessment**	**RGDT**	**FTP**
**1**	*no risk	no risk	normal	altered
**2**	no risk	no risk	normal	normal
**3**	**risk	risk	altered	altered
**4**	risk	no risk	normal	altered
**5**	no risk	risk	normal	altered
**6**	risk	risk	normal	altered
**7**	no risk	no risk	normal	altered
**8**	no risk	risk	normal	altered
**9**	risk	risk	normal	normal
**10**	no risk	risk	normal	normal
**11**	risk	risk	normal	altered
**12**	no risk	risk	normal	normal
**13**	no risk	risk	normal	normal
**14**	risk	no risk	normal	altered

* No risk for CAPD in the QAPAC;

** Risk for CAPD in the QAPAC

## DISCUSSION

This study focuses on the occurrence of CAPD in cases of behavioral dysphonia in children. Studies report that subjects with dysphonia may have difficulty processing the frequency, intensity, and duration of other people’s voices and, consequently, of their own voice, with impairment of vocal self-monitoring^(13,14)^. The strong negative correlation found between the PVSQ and QAPAC reinforces the relationship between CAP and self-perception of voice, suggesting that the inclusion of a simple CAP screening instrument in an evaluation process of dysphonic children can be a useful strategy in the process of clinical evaluation and follow-up.

Correlations were observed between the parental versions and between the self-assessment versions of the two questionnaires, showing a relationship between the perceptions of vocal changes and CAP changes (Table 2). In the QAPAC, the perception of parents was worse than the perception of children. A study that compared questionnaires involving auditory behavior and the diagnostic assessment of CAP in children found a weak to moderate correlation between the questionnaires and the diagnostic tests^(23)^. The authors highlighted disadvantages in applying the questionnaire only to children because of the subjectivity of the answers and the length of the protocols, causing fatigue and, consequently, inaccurate information. Another hypothesis for these findings may be the age difference between the children in our study sample, which ranged from 6 to 10 years. Then, inaccurate answers may be more frequent among younger children and accurate answers among older children.

In a study that applied the QAPAC and compared it with the performance in the ASPA^(10)^, the sample had a mean age of 8.3 years and the answers of children were also compared to the answers of parents, with a worse perception of the children based on the mean score, but this difference was not significant. Another study that investigated the parents’ perception of peripheral hearing complaints of children aged 10 to 13 years disagreed with this finding, as it found a lack of attention from the parents in relation to their children’s complaints^(24)^. On the other hand, a study indicated a significant correlation between the Scale of Auditory Behaviors (SAB) and the CAPD diagnostic tests, indicating reliability in the parents’ perception of their children’s auditory skills^(19)^. Since it is a subjective instrument, divergence between the findings may occur and may be related to the variability of the age group studied and the sample size.

Regarding the distribution of CAPD risk assessed by applying the QAPAC, no significant difference was found between the distribution of children identified at risk and those not at risk for CAPD (Table 1). Ideally, the identification of CAPD is recommended through the application of a battery of behavioral tests that assess auditory skills. The use of a questionnaire would be a possible complementary instrument for diagnosis, as well as electrophysiological tests, contributing to greater diagnostic sensitivity^(2,7)^. In our study, seven children presented risk for CAPD in one of the versions of the QAPAC and, in the diagnostic stage, had alterations in at least one of the temporal auditory processing tests (Table 5). Then, the contribution of the QAPAC is evident in an initial procedure of the assessment of populations seeking care for a non-auditory complaint.

A previous study that analyzed the CAP of 31 dysphonic children and 11 children without voice deviations identified the risk of CAPD in 38.71% of children with dysphonia, when compared to none of the children in the control group^(13)^. However, although not all dysphonic children are at risk for CAPD, screening can support early diagnosis and the voice rehabilitation process, based on a therapeutic plan that includes stimulation of auditory skills, when necessary.

Regarding the PVSQ, although no difference was found between the final score of the protocols in the parental and self-assessment versions, the number of pass/fail was different in the groups, with all parents scoring above the cutoff point, while 70% of the children perceived alterations, also scoring above the cutoff point. Validation studies of this instrument in Brazil^(17)^ and Belgium^(25)^ had higher scores in the self-assessment than in the parental assessment. In our study, the deviated score in the PVSQ in at least one version in the assessment of all children may be related to the sample selection process by active search of children who could have some voice disorder.

The findings regarding the temporal tests showed that only one child presented alterations in the temporal resolution ability, as assessed by the RGDT, and the mean performance data indicated that all of them were above normal (Table 3). A study that analyzed the CAP in children with dysphonia found alterations in the Gaps-In-Noise (GIN) test, which also assesses temporal resolution^(26)^. This ability contributes to the perception of speech and acoustic variations and is related to the minimum time required to resolve or separate acoustic events. In the context of children, this difficulty is related to the process of acquisition and discrimination of phonemes. One hypothesis to be discussed regarding this difference in findings may be related to the differences between the RGDT and GIN tests. The literature discusses differences regarding the parameters of these two tests and the skills involved, and the RGDT may not be considered a purely temporal resolution test, also involving the binaural fusion mechanism, understood as a more complex task. These characteristics sometimes generate more inconsistent answers from children, especially younger ones^(27)^.

The RDGT uses gaps inserted in pure tones and is binaural, while the GIN uses gaps inserted in white noise and can analyze the auditory channels separately. White noise activates several auditory channels at the same time and allows the stimulation of higher levels of the auditory pathway, unlike the pure tone, which evaluates small portions of the auditory pathway and provides spectral clues that can distort the evaluation of the temporal task^(27)^. However, the RGDT is the test on which the AudBility task of temporal resolution ability was based, so it was chosen in order to compare the behavioral evaluation with the online screening. Future studies could apply and compare the RGDT and the GIN tests.

The FPT assesses the temporal ordering ability, which involves the processing of several stimuli in order of occurrence^(28)^. Then, it is important for the recognition of stimuli in the correct order and for the sequencing of the frequency of each sound, participating in the perception of speech, prosody and intonation^(28)^. In our study sample, nine children presented altered performance in at least one ear in one of the stages, and five children presented normal results. In the naming stage, a statistical difference was found between the ears, with better performance of the right ear among right-handed children, and a correlation was observed between the imitation stage of the FPT in the left ear and the QAPAC in the self-assessment version.

In agreement with our study, the literature shows significantly low values in tests that assess the temporal ordering ability of individuals with dysphonia^(14,29)^. In addition, subjects with alterations in this ability have difficulty in the auditory perception of the acoustic parameters of speech (pitch, loudness, and duration), demonstrating the relationship between dysphonia and CAPD^(14)^.

The greater obstacle faced by children in the naming stage of the FPT with the left ear (p<0.05) can be justified by the fact that the right ear transfers information through the crossed auditory pathways directly to the left hemisphere, an associative and dominant area for the processing of verbal language^(30)^. The auditory pathways carry information from the left ear to the right hemisphere, which stabilizes and analyzes the acoustic contour of the sound, but without specificity for verbal language, requiring hemispheric transfer via the corpus callosum to the opposite side (left hemisphere). In children, the corpus callosum is still developing and, therefore, this transfer of information may still be difficult. With the process of neuromaturational development, the corpus callosum reaches its peak of maturation in adolescence and youth and then begins to decline, affecting hearing in the left ear in middle-aged and elderly people^(31)^. Notably, all children who presented this alteration in our study were right-handed.

The results show a sample that, although small, was homogeneous in relation to sex and age distribution. Regarding sex, our data differed from what is reported in the literature, which indicates a higher prevalence of childhood behavioral dysphonia among male subjects, justified by the personality and activities performed by this group, often demanding excessive and inappropriate vocal habits^(13,29)^. Although there is still no consensus, the incidence of CAPD is also indicated as being higher among male children^(3,4)^.

Considering the age variation among the participants (6.3 to 9.3 years), the period of development of central auditory processing is highlighted and, consequently, the differences in expected performance and hemispheric dominance in this age group. Therefore, further studies could use larger samples and specificities at different ages, so that the QAPAC can be used with different populations.

## CONCLUSION

Our findings highlighted the contribution of studied questionnaire in an initial voice assessment protocol applied to children with behavioral dysphonia. In the QAPAC, the perception of parents was worse than the perception of children of their auditory behaviors. Both questionnaires showed changes in results for voice and CAP, indicating that the perception of vocal changes is accompanied by the perception of CAP changes. The relationship between the QAPAC and the FPT in the imitation stage of the left ear shows that the questionnaire is a screening tool to identify changes in auditory skills.
