# Supplementary material for: Use of a self-perception questionnaire for screening auditory abilities in children with behavioral dysphonia
Source: Codas. 2025 Feb 10;37(2):e20230314. doi: 10.1590/2317-1782/e20230314en (PMC11841558; doi:10.1590/2317-1782/e20230314en)
Supplement: Translation

# Translation: Uso de um questionário de autopercepção para triagem das habilidades auditivas em crianças com disfonia comportamental

**DOI:** 10.1590/2317-1782/e20230314pt

**Authors:** Ana Carolina Pinto Lemos, Tamy Nathalia Tanaka, Ana Carolina Constantini, Rebecca Christina Kathleen Maunsell, Maria Isabel Ramos do Amaral

**Affiliations:** 1 Curso de Fonoaudiologia, Departamento de Desenvolvimento Humano e Reabilitação, Faculdade de Ciências Médicas – FCM, Universidade Estadual de Campinas – UNICAMP - Campinas (SP), Brasil.; 2 Curso de Medicina, Departamento de Oftalmologia e Otorrinolaringologia, Faculdade de Ciências Médicas – FCM, Universidade Estadual de Campinas – UNICAMP - Campinas (SP), Brasil.

**Keywords:** Percepção Auditiva, Criança, Triagem, Questionário, Testes Auditivos

## Abstract

**Objetivo:**

Discutir o uso de um questionário de autopercepção das habilidades auditivas em crianças com disfonia comportamental e compará-lo com a percepção de sintomas auditivos e vocais e com o desempenho em testes temporais do processamento auditivo.

**Método:**

Participaram 17 crianças, 6 a 10 anos, com diagnóstico de disfonia comportamental. Perda auditiva periférica, alterações visuais graves, de linguagem ou transtornos do neurodesenvolvimento foram excluídas. O Questionário de Sintomas Vocais Pediátrico (QSV-P) e o Questionário de autopercepção das habilidades auditivas (QAPAC) – inserido no programa de triagem das habilidades auditivas AudBility – ambos em versões autoavaliação e parental, avaliação audiológica básica e os testes temporais Detecção de Intervalo Aleatório (RGDT) e Padrão de Frequência (TPF) foram aplicados. As respostas dos pais e crianças nos questionários foram comparadas e foi mensurado o grau de correlação entre o QAPAC e o QSV-P, bem como entre os questionários e testes temporais.

**Resultados:**

A média de escore no QAPAC na versão de autoavaliação foi 45,5±7,4, com sete (41,2%) crianças abaixo do critério de risco para o Transtorno do processamento auditivo (TPAC). O escore médio da versão parental foi 39,5±10,5, com 11 (64,7%) respostas alteradas. O escore médio dos pais foi pior em relação ao das crianças (p<0,005). Houve forte correlação entre as versões de autoavaliação do QAPAC e QSV-P (r= 0,671) e versões parentais (r= 0,722). Foi observado pior desempenho da orelha esquerda em relação a orelha direita no TPF (p<0,005) e correlação moderada entre o QAPAC e o TPF na orelha esquerda na etapa de imitação (r= 0,597).

**Conclusão**

O uso do QAPAC demonstrou válida contribuição para compor um protocolo inicial de avaliação da voz em crianças com disfonia comportamental.

## INTRODUÇÃO

Sabe-se que o Transtorno do Processamento Auditivo Central (TPAC) é caracterizado pela presença de alteração em uma ou mais habilidades auditivas e apresenta implicações negativas diretas para o desenvolvimento da comunicação e da aprendizagem de uma criança^(1)^. O diagnóstico do TPAC é obtido a partir de uma bateria de testes especiais comportamentais que visam a avaliação de cada uma dessas habilidades, e pode ser complementado por questionários e/ou testes objetivos eletrofisiológicos^(2)^.

Considerando a alta ocorrência do TPAC em crianças em fase escolar^(3)^, protocolos de triagem são de fundamental importância, pois identificam as crianças com risco para esse transtorno. Assim, é feito um encaminhamento assertivo para a avaliação diagnóstica. A partir do diagnóstico, é possível realizar a reabilitação precocemente e minimizar prejuízos futuros^(4)^. Recentemente, o AudBility^(5)^, um programa online de triagem do processamento auditivo, foi desenvolvido e, desde então, estudos têm demonstrado sua viabilidade de aplicação e validação como instrumento de triagem^(6-8)^. O programa apresenta módulos diferentes, dependendo da faixa etária em que será aplicado. Em todos os módulos, há tarefas auditivas e o Questionário de Autopercepção do Processamento Auditivo Central (QAPAC), que pode ser respondido pelo indivíduo que está passando pela triagem, bem como pelos seus pais ou professores^(6-8)^.

Os principais *guidelines* da área recomendam o uso de *checklists* e/ou questionários de autopercepção, tal como o QAPAC, na triagem do PAC, visto que são recursos simples e acessíveis de serem aplicados^(2,9)^. Além disso, os questionários trazem o foco para o indivíduo, detectando as dificuldades enfrentadas no cotidiano por ele e seus cuidadores. Estudo verificou correlação positiva entre o QAPAC e a avaliação simplificada do processamento auditivo (ASPA) em uma amostra de crianças com e sem dificuldades escolares, independente da versão aplicada (autoavaliação ou parental)^(10)^. A ASPA refere-se a uma bateria simples de triagem das habilidades auditivas, comumente realizada por ser um protocolo de baixo custo e rápida aplicação, e verifica as habilidades de localização sonora e memória para sons verbais e não verbais em sequência, por meio de tarefas dicóticas com instrumentos sonoros. Os resultados reforçaram a complementaridade entre o questionário e uma bateria de triagem das habilidades auditivas^(10)^.

Até o momento, o AudBility foi validado como protocolo de triagem em crianças com desenvolvimento típico^(7)^. No estudo de validação, o programa foi aplicado em 154 crianças com bom desempenho escolar. Dessas, 112 também passaram pela avaliação comportamental diagnóstica do Processamento Auditivo Central (PAC). Os resultados demonstraram valores de acurácia que variaram de 54,1% a 84,4% e o AudBility foi considerado eficaz como instrumento de triagem das habilidades auditivas. A partir dessa validação, faz-se necessário estudar sua contribuição quando aplicado a outras populações. Entre as alterações associadas ao TPAC, destaca-se a disfonia comportamental, decorrente do uso inadequado da voz e definida como dificuldade na emissão vocal que impede a produção natural da voz, acarretando prejuízos à comunicação do indivíduo^(11)^.

Evidências sugerem que queixas vocais podem estar relacionadas a dificuldades do PAC e apontam alterações nas habilidades auditivas temporais de crianças disfônicas^(12,13)^. As habilidades auditivas temporais, principalmente as de ordenação e resolução temporal, estão envolvidas no processamento da voz, e sabe-se que um indivíduo com dificuldade de processar a frequência, a intensidade e a duração da fala é prejudicado no automonitoramento e, consequentemente, na produção vocal^(14,15)^.

Acredita-se que a triagem do processamento auditivo de crianças com disfonia comportamental possibilitaria a identificação de maneira simples e rápida daquelas que poderiam se beneficiar da avaliação diagnóstica completa do PAC. O objetivo deste estudo foi descrever e analisar o uso do questionário de Autopercepção das Habilidades Auditivas aplicado em crianças com disfonia comportamental e compará-lo com a percepção de sintomas auditivos e vocais, bem como com o desempenho em testes temporais do processamento auditivo.

## MÉTODO

### Tipo, local do estudo e aspectos éticos

Trata-se de um estudo quantitativo, descritivo, prospectivo e de corte transversal, aprovado pelo Comitê de Ética em Pesquisa, sob o parecer 4.793.214. Essa pesquisa faz parte de um projeto maior intitulado “Aplicabilidade do programa de triagem das habilidades auditivas - AudBility - em crianças com disfonia comportamental”. A coleta de dados ocorreu nos Ambulatórios de Otorrinolaringologia, Voz e Audiologia da Instituição em que a pesquisa foi realizada. A participação foi voluntária, sendo que o Termo de consentimento Livre e Esclarecido (TCLE) foi assinado pelos pais e o Termo de Assentimento Livre e Esclarecido (TALE) foi assinado pelas crianças.

### Participantes e critérios de seleção

Os sujeitos foram selecionados a partir de um levantamento da demanda de crianças com disfonia comportamental, na faixa etária de 6 a 10 anos, de ambos os sexos, com entrada no serviço pelo Ambulatório de Pronto Atendimento Vocal (PAV) da Instituição e de uma Unidade Básica de Saúde do município.

Foram incluídas crianças falantes nativas do português brasileiro, com diagnóstico confirmado de disfonia comportamental a partir de avaliação médica e fonoaudiológica. Foram excluídas crianças que apresentassem perda auditiva periférica, histórico de otites de repetição, alterações visuais severas, alterações cognitivas/síndromes e/ou transtornos do neurodesenvolvimento ou de linguagem e alterações anatômicas da laringe. Também foram excluídas crianças que já haviam feito fonoterapia direcionada para aspectos vocais e/ou de linguagem e que possuíam dificuldades escolares atestadas pelo professor e confirmada pelo histórico levantado com os pais e/ou responsáveis.

### Procedimentos

A coleta de dados foi feita em duas etapas. A primeira etapa consistiu na confirmação do diagnóstico de disfonia comportamental e do funcionamento normal da audição periférica, por meio da avaliação otorrinolaringológica e fonoaudiológica, além da verificação do bom desempenho escolar. Esses procedimentos estão descritos a seguir:

- Avaliação fonoaudiológica da voz: foi realizada gravação da voz em cabine acústica e feito julgamento perceptivo-auditivo da qualidade vocal por dois juízes neutros, fonoaudiólogos com experiência na área de voz. Para tal avaliação foi utilizando o Protocolo CAPE-V – escala analógico-visual^(16)^. Como parte da avaliação fonoaudiológica da voz e também para a coleta de dados, foi aplicado o Questionário de Sintomas Vocais Pediátrico (QSV-P)^(17)^, na versão respondida pela criança (autoavaliação) e parental. No QSV-P, as questões envolvem o uso da voz e os impactos das alterações vocais. São contemplados os quatro domínios da voz: cantada, falada, gritada e projetada, e os participantes respondem sobre a frequência de cada situação apresentada (0 = nunca, 1 = às vezes, 2 = quase sempre e 3 = sempre). O máximo do escore é de 38 pontos, o que indica alto impacto. As notas de corte que indicam alterações vocais são: 2.1 para a versão parental e 7.6 para a versão autoavaliação. Os dados obtidos no QSV-P, além de comporem a avaliação vocal, foram analisados e comparados com os resultados encontrados no QAPAC e com os testes comportamentais do processamento auditivo temporal.
- Avaliação otorrinolaringológica: realizada por um otorrinolaringologista especializado em atendimento pediátrico. Na avaliação médica foram coletados dados sobre queixas respiratórias e audiológicas e realizado o exame físico otorrinolaringológico, incluindo videonasofibrolaringoscopia.
- Avaliação Audiológica Básica: foi composta pela meatoscopia, anamnese, Audiometria Tonal liminar, Logoaudiometria e Imitanciometria para confirmar a audição dentro dos padrões da normalidade (OMS, 2014) e uma curva timpanométrica do tipo A^(18)^. As crianças com excesso de cerume foram encaminhadas para remoção e posteriormente reconvocadas. Os testes foram realizados com o audiômetro GSI AudioStar Pro em cabina acústica e com o imitanciômetro Interacoustics AT235, devidamente calibrados.
- Confirmação do bom desempenho escolar: foi enviado um questionário para ser respondido pelo professor responsável pela criança, a respeito do rendimento escolar do aluno, percepção sobre o comportamento auditivo e atencional e relacionamento com os pares. Esse questionário foi entregue aos pais no primeiro dia e trazido respondido pelo professor no segundo dia de coleta de dados. Além do questionário respondido pelo professor, também foi considerada a ausência de queixas escolares relatada pelos pais ou responsáveis no momento da anamnese.

A segunda etapa, realizada em outro dia, consistiu na aplicação do questionário do programa AudBility e dos testes diagnósticos do processamento auditivo temporal em cabina acústica.

O programa AudBility é composto pelo Questionário de autopercepção das habilidades auditivas (QAPAC) e uma bateria de tarefas auditivas. No presente estudo, analisaram-se apenas as respostas do QAPAC, detalhado a seguir:

- Questionário de autopercepção das habilidades auditivas – QAPAC^(10)^: O QAPAC foi elaborado com base no instrumento “*Scale of Auditory Behaviors*” – SAB^(19)^ e publicado em 2018^(10)^, nas versões autoavaliação e parental, disponíveis no Quadro 1. São apresentadas 12 perguntas diretas que dizem respeito aos comportamentos auditivos em ambientes silencioso e ruidoso, em linguagem compreensiva para os participantes, que respondem com qual frequência determinado comportamento auditivo ocorre em cada situação. Anteriormente à pergunta, há uma situação-exemplo que contextualiza a situação, para facilitar o entendimento. Para cada resposta há uma pontuação, seguindo a escala Likert: sempre (1), frequentemente (2), algumas vezes (3), raramente (4) e nunca (5). O resultado é calculado através da soma das respostas e o escore pode variar de 12 a 60 pontos. Escores abaixo de 45 pontos foram considerados de risco para ocorrência do TPAC^(6,7,19)^.

**Quadro 1:** Questões do QAPAC - versões autoavaliação e parental

**Questionário criança**	**Questionário pais**
**Você está em uma sala de aula ou em um ambiente em que tem pessoas conversando**	**Quando seu (a) filho (a) está em um ambiente em que tem pessoas conversando**
**1. Você tem dificuldade para escutar ou entender o que a professora está falando?**	**1. Ele (a) tem dificuldade para escutar ou entender o que as pessoas estão falando?**
**A professora ou uma pessoa estão falando muito rápido com você**	**Se você fala muito rápido com seu (a) filho (a)**
**2. Você tem dificuldade para entender o que a professora falou?**	**2. Ele (a) tem dificuldade para entender o que foi falado?**
**A professora ou uma pessoa estão dando instruções (explicações) faladas para você**	**Quando você dá instruções (explicações) faladas para seu (a) filho (a)**
**3. Você tem dificuldade para seguir as instruções faladas?**	**3. Ele (a) tem dificuldade para seguir as instruções faladas?**
**A professora ou uma pessoa estão falando com você em um ambiente silencioso**	**Se você está falando com seu (a) filho (a) em um ambiente silencioso**
**4. Você tem dificuldade para escutar e entender claramente as palavras sem trocar nenhuma letra?**	**4. Ele (a) tem dificuldade para escutar e entender claramente as palavras sem trocar nenhuma letra?**
**Quando a professora ou um amigo estão falando com você**	**Quando você está falando com seu (a) filho (a)**
**5. Você tem a sensação que às vezes você ouve bem e às vezes não?**	**5. Você tem a sensação de que às vezes ele (a) ouve bem e às vezes não?**
**Você está em sala de aula ou no pátio da escola e alguém chama seu nome**	**Quando seu (a) filho (a) é chamado pelo nome em um local amplo**
**6. Você tem dificuldade para perceber de onde vem o som?**	**6. Ele tem dificuldade para perceber de onde vem o som?**
**A professora ou uma pessoa estão falando com você**	**Quando você está falando com seu (a) filho (a)**
**7. Você pede para repetir o que foi falado?**	**7. Ele (a) pede para repetir o que foi falado?**
**Você está na sala de aula**	**Quando seu (a) filho (a) está em casa ou em outros ambientes**
**8. Você fica distraído com facilidade?**	**8. Ele fica distraído com facilidade?**
**No ano passado na escola**	**No ano passado**
**9. Você teve dificuldades para aprender?**	**9. Seu (a) filho (a) teve dificuldade para aprender?**
**Você está fazendo uma atividade**	**Quando seu (a) filho (a) está fazendo uma atividade escolar**
**10. Você tem dificuldade para ficar atento?**	**10. Ele (a) tem dificuldade para ficar atento?**
**Quando você está em sala de aula ou em casa**	**Quando seu (a) filho (a) está em casa**
**11. As pessoas falam que você é sonhador ou desatento?**	**11. Você acha que ele (a) é sonhador ou desatento?**
**Quando você está na escola ou em casa**	**Quando seu filho (a) está em casa**
**12. Você é desorganizado?**	**12. Ele (a) é desorganizado?**

O programa apresenta inicialmente uma tela de apresentação do questionário e das opções de resposta e pictogramas relacionados a cada uma das opções de resposta, de modo a facilitar a compreensão da criança, conforme demonstrado na Figura 1. A Figura 2 ilustra a tela referente à primeira pergunta do QAPAC, juntamente com as opções de resposta da criança.

**Figura 1:** Tela de treino do QAPAC

**Figura 2:** Tela do programa AudBility, primeira questão do QAPAC

Para a aplicação com a criança, o pesquisador signatário deste estudo leu as perguntas juntamente com a criança para auxiliar na realização da tarefa. A criança apontou na tela do computador sua resposta e o pesquisador selecionou a opção desejada.

- Avaliação Comportamental do Processamento Auditivo Temporal: Os testes comportamentais do processamento auditivo temporal foram realizados em cabina acústica, com o audiômetro GSI AudioStar Pro e fones supraurais, devidamente calibrados. Os testes serão descritos a seguir:
  - Teste de Detecção de Intervalo Aleatório - *Random Gap Detection Test* – RGDT^(20)^: Avalia a habilidade de resolução temporal em uma tarefa binaural em intensidade de 50dBNS (nível de sensação). São apresentados pares de tons puros nas frequências de 500, 1000, 2000 e 4000 Hz, com intervalos/gaps entre os dois tons, de duração variável e dispostos aleatoriamente. Os gaps variam entre intervalos de 0, 2, 5, 10, 15, 20, 25, 30 e 40 milissegundos (ms) e o teste apresenta uma faixa de treino. As crianças foram orientadas a responder gestualmente se ouviram/perceberam um ou dois tons, sendo possível observar se elas notam ou não a presença do gap interestímulos. Determinou-se o limiar de detecção de gap, ou seja, o menor intervalo de tempo percebido. O limiar foi calculado individualmente para cada frequência, bem como a média aritmética de resultados nas quatro frequências avaliadas. O critério de normalidade considera limiar menor ou igual a 15ms para crianças de seis anos e menor ou igual a 10ms para crianças com sete anos ou mais^(21)^.
  - Teste de Padrão de Frequência: Avalia a habilidade de ordenação temporal em uma tarefa monoaural, na intensidade de 40dBNS (nível de sensação). São 30 sequências de três tons que se diferem pela frequência, em duas etapas diferentes: a de imitação e a de nomeação dos sons (grave ou agudo). Aplicou-se a versão Audtec de St Louis^(22)^: Nesta versão, é apresentada uma lista com 30 sequências por orelha em cada etapa, sendo combinações de tons puros que se diferem pela frequência - grave: 880Hz / agudo 1430Hz; com duração de 500ms. A porcentagem de acertos foi calculada por orelha e o critério de normalidade considera porcentagem maior ou igual a 60% para crianças de seis e sete anos, e maior ou igual a 81,5% para crianças de oito anos ou mais.

### Análise dos resultados

A análise estatística foi realizada utilizando-se os softwares SPSS V20, Minitab 16 e Excel Office 2010. A descrição das variáveis foi apresentada a partir de estatística descritiva e inferencial.

O teste de Igualdade de Duas Proporções analisou a amostra em relação ao sexo e a distribuição da frequência relativa (percentual) do resultado “passou ou falhou” nos questionários. O teste de Wilcoxon comparou o desempenho entre as respostas da criança (autoavaliação) e pais (parental) em cada um dos questionários aplicados (QAPAC e QSV-P) e o desempenho entre a orelha direita e a orelha esquerda no Teste de Padrão de Frequência. A Correlação de *Spearman* mensurou o grau de correlação entre o QSV-P e o QAPAC, bem como entre os questionários e os testes temporais (TPF e RGDT). Para os coeficientes de correlação (r) foi assumida a seguinte escala: |0,10| a |0,40| - correlação fraca; |0,40| a |0,60| - correlação moderada; |0,60| a |1,00| - correlação forte. O intervalo de confiança adotado foi de 95%, considerando-se, então, o nível de significância de 5%. Os achados significantes (p<0,05) foram destacados com asterisco (*) e em negrito nas Tabelas apresentadas.

## RESULTADOS

A amostra foi composta por 17 crianças, com idades entre 6 e 10 anos, sendo a média 7,8+1,5 anos. Houve baixa variabilidade quanto a idade (CV = 19%) e homogeneidade entre a distribuição dos sexos (p valor = 0,003), sendo sete (41,2%) meninas e 10 (58,8%) meninos. Apenas duas crianças (11,76%) eram canhotas.

Em relação ao desempenho da amostra nos questionários, a média no QAPAC foi de 45,5 na versão autoavaliação e de 39,5 na versão parental. A comparação entre as respostas das versões mostrou pior percepção dos pais quanto ao comportamento auditivo da criança (p=0,016). Além disso, a média dos escores obtidos na aplicação com os pais foi abaixo da nota de corte (<45 pontos), sugerindo risco para a ocorrência do Transtorno do Processamento Auditivo (TPAC). No QSV-P, a média está acima da nota de corte em ambas as versões, sendo 11,1 na autoavaliação e 12 na parental e, neste instrumento, tal dado indica a percepção de sintomas vocais. Não houve diferença significativa na comparação entre as respostas das versões do QSV-P.

Houve diferença significativa entre o número de crianças que passaram e falharam no QSV-P (Tabela 1), considerando tanto a versão autoavaliação quanto a versão parental.

**Tabela 1:** Distribuição da amostra nas duas versões dos questionários de Autopercepção do Processamento Auditivo (QAPAC) e Questionário de Sintomas Vocais Pediátricos (QSV-P)

		**N**	**%**	**p-valor**
**QAPAC autoavaliação**	Falhou	7	41,20%	0,303
	Passou	10	58,80%	
**QAPAC parental**	Falhou	11	64,70%	0,086
	Passou	6	35,30%	
**QSV-P autoavaliação**	Falhou	12	70,60%	**0,016** *
	Passou	5	29,40%	
**QSV-P parental**	Falhou	16**	100%	<0,001*
	Passou	0	0%	

* Teste de Igualdade de Duas Proporções;

** Um pai/responsável não respondeu ao questionário

Na correlação entre o escore médio da amostra obtido no QAPAC em relação e o escore médio obtido no QSV-P (Tabela 2), observou-se correlação forte negativa entre as versões dos questionários respondidas pelas crianças e entre as versões respondidas pelos pais, ou seja, quanto maior for a frequência de sintomas vocais, maior é o risco para o TPAC.

**Tabela 2:** Correlação entre os escores obtidos no Questionário de Autopercepção do Processamento Auditivo (QAPAC) e Questionário de Sintomas Vocais Pediátricos (QSV-P) - versões autoavaliação e parental

		**QSV-P (criança)**	**QSV-P(adulto)**
**QAPAC (criança)**	**Corr** ** **(r)**	-0,671	-0,170
	**P-valor**	0,003*	0,529
**QAPAC (adulto)**	**Corr (r)**	-0,387	-0,722
	**P-valor**	0,125	**0,002** *

** Corr = correlação;

* Correlação de Spearman

Das 17 crianças da amostra, 14 compareceram no segundo dia de coleta de dados para a avaliação comportamental do processamento auditivo temporal. Dos sujeitos avaliados no RGDT, apenas um (7,14%) apresentou resultado alterado. No TPF, nove (64,28%) crianças apresentaram desempenho alterado em ao menos uma orelha de uma das etapas e cinco (35,71%) crianças apresentaram resultados normais. Na Tabela 3 é possível observar o desempenho médio da amostra no TPF e no RGDT, além da comparação entre o desempenho médio das orelhas direita e esquerda no TPF. Foi observada diferença estatística na etapa de nomeação, com melhor desempenho da orelha direita (p<0,005).

**Tabela 3:** Desempenho da amostra no Teste de Padrão de Frequência (TPF) e no teste de Detecção de Intervalos Aleatórios (RGDT) e comparação entre as orelhas no TPF

**Processamento Auditivo Temporal**		**N**	**Média**	**Mediana**	**Desvio Padrão**	**IC**	**OE × OD**
**TPF imitação**	**OD**	14	74,5	88	28,1	14,7	0,374
	**OE**	14	77,4	80	21,3	11,1	
**TPF nomeação**	**OD**	14	64,1	62	16,8	8,8	**0,026** *
	**OE**	14	56,9	52	21,6	11,3	
**RGDT**		14	4,8	4	3	1,7	-

* Teste de Wilcoxon

Legenda: IC = intervalo de confiança; OE = orelha esquerda; OD = orelha direita

A Tabela 4 apresenta as correlações entre os questionários estudados (QAPAC e QSV-P) em relação aos dois testes do processamento auditivo temporal. Houve correlação moderada e positiva entre o QAPAC na versão de autoavaliação e o TPF, considerando a etapa de imitação da orelha esquerda (p<0,005).

**Tabela 4:** Correlação entre o questionário de Autopercepção do Processamento Auditivo Central (QAPAC), Questionário de Sintomas Vocais Pediátricos (QSV-P) e os testes do processamento auditivo temporal

**Processamento Auditivo Temporal**		**QSV-P (criança)**	**QSV-P (adulto)**	**QAPAC (criança)**	**QAPAC (adulto)**
**RGDT**	Corr (r)	0,277	0,451	-0,093	-0,211
	P-valor	0,384	0,164	0,774	0,510
**TPF OD (nomeação)**	Corr (r)	-0,150	0,448	0,124	-0,354
	P-valor	0,609	0,124	0,673	0,214
**TPF OE (nomeação)**	Corr (r)	-0,209	0,532	0,254	-0,389
	P-valor	0,472	0,061	0,380	0,170
**TPF OD (imitação)**	Corr (r)	-0,214	0,351	0,506	-0,145
	P-valor	0,463	0,240	0,065	0,621
**TPF OE (imitação)**	Corr (r)	-0,509	0,055	0,597	0,179
	P-valor	0,063	0,858	**0,024** *	0,541

* Correlação de Spearman

Legenda: TPF = Teste de Padrão de Frequência; OD = orelha direita; OE = orelha esquerda

Por fim, a tabela 5 mostra que, das 14 crianças avaliadas nos testes do processamento auditivo temporal, 9 obtiveram alteração. Das 9 crianças, 7 apresentaram risco para o TPAC em pelo menos uma das versões do QAPAC.

**Tabela 5:** Condições individuais da amostra em relação aos testes do processamento auditivo temporal e no Questionário de Autopercepção do Processamento Auditivo Central (QAPAC)

**Sujeitos**	**QAPAC autoavaliação**	**QAPAC parental**	**RGDT**	**TPF**
**1**	*não risco	não risco	normal	alterado
**2**	não risco	não risco	normal	normal
**3**	**risco	risco	alterado	alterado
**4**	risco	não risco	normal	alterado
**5**	não risco	risco	normal	alterado
**6**	risco	risco	normal	alterado
**7**	não risco	não risco	normal	alterado
**8**	não risco	risco	normal	alterado
**9**	risco	risco	normal	normal
**10**	não risco	risco	normal	normal
**11**	risco	risco	normal	alterado
**12**	não risco	risco	normal	normal
**13**	não risco	risco	normal	normal
**14**	risco	não risco	normal	alterado

* Não há risco para o TPAC no QAPAC;

** Há risco para o TPAC no QAPAC

## DISCUSSÃO

A temática desta pesquisa é centrada na ocorrência do TPAC em quadros de disfonia infantil. Estudos relatam que sujeitos com disfonia podem apresentar dificuldade em processar a frequência, a intensidade e a duração da voz das outras pessoas e, consequentemente, da própria voz, sendo prejudicados no automonitoramento vocal^(13,14)^. A correlação negativa forte encontrada entre os questionários QSV-P e QAPAC reforça a relação entre o PAC e a autopercepção da voz, sugerindo que a inclusão de um instrumento simples de triagem do PAC em um processo de avaliação da criança disfônica pode ser uma estratégia útil no processo de avaliação e seguimento clínico.

As correlações observadas ocorreram tanto entre as versões parentais quanto entre as versões de autoavaliação dos dois questionários, mostrando que há uma relação entre as percepções de alterações vocais e de alterações do PAC (Tabela 2). No QAPAC, a percepção dos pais foi pior do que a percepção das crianças. Estudo que comparou questionários envolvendo o comportamento auditivo e a avaliação diagnóstica do PAC em crianças encontrou correlação fraca a moderada entre os questionários e os testes diagnósticos^(23)^. Assim, os autores apontaram desvantagens na aplicação do questionário apenas em crianças, que podem se dar pela subjetividade das respostas e a extensão dos protocolos, trazendo cansaço e, consequentemente, informações imprecisas. Uma outra hipótese para esses achados pode ser a diferença de idade entre as crianças da amostra do nosso estudo, que variou de 6 a 10 anos. Assim, crianças menores poderiam ter mais imprecisão nas respostas e crianças maiores podem ser mais precisas.

No estudo que aplicou o QAPAC e comparou com o desempenho na ASPA^(10)^, a amostra apresentou média de idade de 8,3 anos e as respostas das crianças também foram comparadas com as respostas dos pais, com pior percepção das crianças a partir do escore médio, porém tal diferença não foi significativa. Outro estudo que realizou investigação acerca da percepção dos pais sobre as queixas de audição periféricas dos filhos de 10 a 13 anos divergiu desse achado, pois encontrou falta de atenção dos pais em relação às queixas dos filhos^(24)^. Por outro lado, uma pesquisa apontou correlação significativa entre o *Scale of Auditory Behaviors* (SAB) e os testes diagnósticos do TPAC, indicando confiabilidade na percepção dos pais sobre as habilidades auditivas dos filhos^(19)^. Tratando-se de um instrumento subjetivo, a divergência entre os achados pode ocorrer e pode estar relacionada à variabilidade da faixa etária estudada e ao número da amostra.

Com relação à distribuição do risco de TPAC avaliado a partir da aplicação do QAPAC, não foi encontrada diferença significativa entre a distribuição de crianças identificadas com risco e sem risco para o TPAC (Tabela 1). A identificação do TPAC, idealmente, é recomendada a partir da aplicação de uma bateria composta por testes comportamentais que avaliam as habilidades auditivas. O uso de um questionário seria uma possibilidade de instrumento complementar ao diagnóstico, assim como os testes eletrofisiológicos, contribuindo com maior sensibilidade diagnóstica^(2,7)^. Em nosso estudo, sete crianças apresentaram risco para o TPAC em uma das versões do QAPAC e, na etapa de diagnóstico, tiveram alteração em ao menos um dos testes do processamento auditivo temporal (Tabela 5). Assim, evidencia-se a contribuição do QAPAC em um procedimento inicial para compor uma avaliação de populações que buscam atendimento para uma queixa principal que não seja a auditiva.

Estudo anterior que analisou o PAC de 31 crianças disfônicas e 11 crianças sem alterações vocais identificou o risco de TPAC em 38,71% das crianças com disfonia, em comparação a nenhuma criança do grupo controle^(13)^. No entanto, apesar de nem todas as crianças disfônicas apresentarem risco para TPAC, uma triagem pode beneficiar o diagnóstico precoce e o processo de reabilitação vocal, a partir de um planejamento terapêutico que inclua a estimulação de habilidades auditivas, quando necessário.

Com relação ao QSV-P, embora não tenha sido encontrada diferença entre o escore final dos protocolos nas versões parental e autoavaliação, a distribuição de passou/falhou foi diferente nos grupos, sendo que todos os pais pontuaram acima do ponto de corte, enquanto 70% das crianças perceberam alterações, pontuando também acima da nota de corte. Os estudos de validação deste instrumento no Brasil^(17)^ e na Bélgica^(25)^ encontraram maiores escores do protocolo na autoavaliação do que na avaliação parental. No presente estudo, a ocorrência de escore alterado no QSV-P em pelo menos uma versão na avaliação de todas as crianças pode estar relacionada ao processo de seleção da amostra por busca ativa das crianças que pudessem ter alguma alteração vocal.

Os achados em relação aos testes temporais demonstraram que apenas uma criança apresentou alteração na habilidade de resolução temporal, avaliada pelo teste RGDT, sendo que o dado médio de desempenho indicou que todas estavam acima da normalidade (Tabela 3). Estudo que analisou o PAC em crianças com disfonia verificou alteração no teste Gaps-In-Noise (GIN), que também avalia a resolução temporal^(26)^. Tal habilidade contribui com a percepção de fala e variações acústicas, estando relacionada ao tempo mínimo necessário para solucionar ou separar acontecimentos acústicos. No contexto infantil, essa dificuldade se relaciona com o processo de aquisição e discriminação de fonemas. Uma hipótese a ser discutida quanto a essa diferença de achado pode estar relacionada às diferenças entre os testes RGDT e GIN. A literatura discute diferenças em relação aos parâmetros desses dois testes e habilidades envolvidas, sendo possível que o RGDT não seja considerado um teste puramente de resolução temporal, envolvendo também o mecanismo de fusão binaural, entendido como uma tarefa mais complexa. Essas características, por vezes, geram respostas mais inconsistentes das crianças, especialmente as menores^(27)^.

O RDGT utiliza gaps inseridos em tons puros e é binaural, enquanto o GIN utiliza gaps inseridos em ruído branco e pode analisar as orelhas separadamente. O ruído branco ativa diversos canais auditivos ao mesmo tempo e permite a estimulação de níveis mais altos da via auditiva, diferenciando-se do tom puro, que avalia pequenas porções da via auditiva e fornece pistas espectrais que podem distorcer a avaliação da tarefa temporal^(27)^. Entretanto, o RGDT é o teste no qual a tarefa de habilidade de resolução temporal presente no AudBility foi baseada e, para que fosse possível comparar a avaliação comportamental com a triagem online, foi escolhido este teste. Em próximos estudos, recomenda-se que o RGDT e o GIN sejam aplicados e comparados.

O TPF avalia a habilidade de ordenação temporal, que envolve o processamento de diversos estímulos em uma ordem de ocorrência^(28)^. Dessa forma, ela é importante para o reconhecimento dos estímulos em ordem correta e para a sequencialização da frequência de cada som, participando da percepção de fala, prosódia e entonação^(28)^. Na amostra estudada, nove crianças apresentaram desempenho alterado em ao menos uma orelha de uma das etapas e cinco crianças apresentaram resultados normais. Na etapa de nomeação, foi encontrada diferença estatística entre as orelhas, com melhor desempenho da orelha direita entre as crianças destras e correlação entre a etapa de imitação do TPF na orelha esquerda e o QAPAC na versão autoavaliação.

Em concordância com a nossa pesquisa, a literatura apresenta valores significativamente baixos em testes que avaliam a habilidade de ordenação temporal de pessoas com disfonia^(14,29)^. Além disso, os sujeitos com alteração em tal habilidade possuem dificuldade na percepção auditiva dos parâmetros acústicos da fala (*pitch*, *loudness* e duração), evidenciando a relação entre a disfonia e o TPAC^(14)^.

A maior dificuldade encontrada pelas crianças na etapa de nomeação do TPF na orelha esquerda (p<0,05) pode ser justificada pela vantagem que a orelha direita possui de ter suas informações transferidas, através das vias auditivas cruzadas, diretamente para o hemisfério esquerdo, área associativa e dominante para o processamento da linguagem verbal^(30)^. As vias auditivas levam a informação da orelha esquerda para o hemisfério direito, que estabiliza e analisa o contorno acústico do som, porém sem especificidade para a linguagem verbal, sendo necessária a transferência hemisférica via corpo caloso ao lado oposto (hemisfério esquerdo). Em crianças, o corpo caloso ainda está em desenvolvimento e, por isso, pode haver uma dificuldade nessa transferência de informações. Com o processo de desenvolvimento neuromaturacional, o corpo caloso atinge seu pico maturacional na adolescência e juventude e, então, passa a decair, ocasionando piora na escuta da orelha esquerda em pessoas de meia-idade e idosos^(31)^. Reforça-se que todas as crianças que apresentaram essa alteração no presente estudo eram destras.

Os resultados analisados apresentam uma amostra que, apesar de pequena, foi homogênea em relação à distribuição do sexo e à idade. Com relação ao sexo, esse dado divergiu do que é posto pela literatura, que aponta maior prevalência de disfonia comportamental infantil em meninos, justificada pela personalidade e atividades exercidas por este grupo, que muitas vezes demandam hábitos vocais excessivos e inadequados^(13,29)^. Mesmo que ainda não haja um consenso, a incidência de TPAC é também apontada como sendo maior em meninos^(3,4)^.

Tendo em vista a variação de idade entre os participantes (de 6,3 e 9,3 anos), ressalta-se o período de desenvolvimento do processamento auditivo central e, consequentemente, as diferenças na expectativa de desempenho e na dominância hemisférica presentes dentro dessa faixa etária. Assim, são sugeridos estudos com amostras maiores e especificidades para as diferentes idades, para que o QAPAC possa ser usado com diferentes populações.

## CONCLUSÃO

Os achados evidenciaram a contribuição do questionário estudado em um protocolo de avaliação inicial de voz em crianças com disfonia comportamental. No QAPAC, identificou-se que a percepção dos pais foi pior do que a percepção das crianças sobre seus comportamentos auditivos. Ambos os questionários revelaram alteração em seus resultados, tanto em relação à voz quanto ao PAC, indicando que percepção de alterações vocais estão acompanhadas de percepção de alterações de PAC. A relação entre o QAPAC e o TPF na etapa de imitação da orelha esquerda aponta o questionário como um instrumento de rastreio para alteração nas habilidades auditivas.
